# Microscopic Receding Contact Line Dynamics on Pillar and Irregular Superhydrophobic Surfaces

**DOI:** 10.1038/srep08384

**Published:** 2015-02-11

**Authors:** Yong Han Yeong, Athanasios Milionis, Eric Loth, Ilker S. Bayer

**Affiliations:** 1 Department of Mechanical and Aerospace Engineering, University of Virginia, Charlottesville, Virginia 22904, USA; 2 Smart Materials, Central Laboratories, Istituto Italiano di Tecnologia, Genoa, 16162, Italy

## Abstract

Receding angles have been shown to have great significance when designing a superhydrophobic surface for applications involving self-cleaning. Although apparent receding angles under dynamic conditions have been well studied, the microscopic receding contact line dynamics are not well understood. Therefore, experiments were performed to measure these dynamics on textured square pillar and irregular superhydrophobic surfaces at micron length scales and at micro-second temporal scales. Results revealed a consistent “slide-snap” motion of the microscopic receding line as compared to the “stick-slip” dynamics reported in previous studies. Interface angles between 40–60° were measured for the pre-snap receding lines on all pillar surfaces. Similar “slide-snap” dynamics were also observed on an irregular nanocomposite surface. However, the sharper features of the surface asperities resulted in a higher pre-snap receding line interface angle (~90°).

Inspired by organisms in nature such as the lotus leaf^1^ and water strider legs^2^, superhydrophobic surfaces feature remarkable water repellency which are widely known to be governed by a combination of roughness at the micro/nano scale and low surface energy^3^,^4^,^5^. These two parameters reduce the area of contact between the solid-liquid interface as well as its molecular attraction to trap pockets of air between the surface asperities, hence resulting in a Cassie-Baxter wetting state^6^. This is in contrast to the Wenzel wetting state^7^, whereby a liquid fully penetrates into areas between the surface features.

Under the Cassier-Baxter^6^ and Wenzel^7^ wetting models, the affinity of water to a surface can be quantified by the contact angle (CA), an equilibrium angle characterizing the three phase contact line of water, surface and air. However, it has been comprehensively agreed by researchers that the sole use of CA is insufficient to describe the wettability of the surface^8^,^9^,^10^,^11^,^12^, especially within the context of practical applications of superhydrophobic surfaces such as self-cleaning. This was confirmed by Wang *et al.*^13^ and Bhushan *et al.*^14^ who reported the possibility of various non-wetting states with scenarios such as the Cassie, Wenzel, “gecko” and “rose petal effect”. In all these cases, CA's remain high. However, the mobility of a water drop on these surfaces varies. For example, a surface synthesized by Bhushan *et al*.^14^ depicting a “petal effect” had a high CA of 152° but also displayed strong adhesion, to the point where a drop would stick to the surface even when tilted at 90 degrees. It is therefore clear that additional parameters would be required to quantify the state of superhydrophobicity of a surface.

To accomplish this, dynamic angles such as the advancing and receding angles were prescribed^15^,^16^,^17^,^18^. Consider the case where a drop rolls on a tilted superhydrophobic surface. For the drop to move forward, the leading edge of the droplet has to advance, creating an angle on the three-phase contact line called the advancing angle. On the other hand, the trailing edge of the drop retreats from the surface to form the receding angle. Both the advancing and receding contact line motions are highly dynamic, transitioning from one metastable state to another and have been extensively studied using theoretical^16^,^17^,^18^,^19^, experimental^9^,^20^,^21^,^22^,^23^,^24^,^25^,^26^,^27^,^28^,^29^ and computational methods^30^,^31^,^32^. Researchers have observed that the advancing three-phase contact line on superhydrophobic surfaces consisting of pillar geometries does not move, but rather descends upon its adjacent pillar to wet the top of the pillar surface^17^,^18^,^19^,^20^,^21^,^22^,^30^. The receding contact line on the other hand is forced to detach from one pillar to another in a discrete fashion, creating a pinning-depinning motion. Although the occurrence of this receding contact line detachment has been well documented^19^,^20^,^21^,^22^,^23^,^24^,^25^,^26^,^27^, questions still remain on the precise dynamics and degree in which the receding line disjoins from one pillar to another. In fact, researchers have hypothesized conflicting scenarios for the receding mechanism. Gao and McCarthy^23^ suggested that the contact line remains pinned on the entire pillar top until it instantly detaches to move towards the next pillar, after which it relaxes. Dorrer and Rühe^21^ proposed that the contact line remains pinned only on the very edge of the pillar before following a similar detachment and relaxation motion. Krumpfer *et al.*^20^ hypothesized that the receding line would detach in a near vertical (tensile) manner to rupture the capillary bridge before leaving small sessile droplets on the de-wetted posts. However, Extrand^18^ speculated that the contact line would travel along the pillar top in a horizontal direction prior to pining at the edge and detachment. The inconsistency in these hypotheses signified the need for experimental measurements to validate these predictions, a necessity that was acknowledged by Patankar^19^.

This was recently accomplished by Paxson and Varanasi^33^, who were able to experimentally measure the dynamic behavior and angle of the microscopic receding contact lines along with its capillary bridge. A self-similar depinning mechanism of the drop at different length scales was also observed. However, due to poor temporal resolution, they were unable to resolve the depinning dynamics of the contact line. Hence, the exact dynamic behavior of the receding line remains unknown. Understanding the depinning mechanisms would not only improve our fundamental comprehension of adhesion and wettability of superhydrophobic surfaces at the microscopic level but would also have profound implications on practical applications. For example, recent research in the application of superhydrophobic surfaces in reducing surface ice adhesion and marine bio-fouling have resulted in the discovery of the receding angle as the controlling wetting parameter towards ice and fouling resistance^34^,^35^,^36^. Therefore, the understanding of receding line dynamics at the microscopic level could potentially lead to a more successful optimization and implementation of superhydrophobic surfaces in these applications.

In this study, we present to the best of our knowledge, a first experimental investigation to measure the microscopic receding contact line dynamics of superhydrophobic surfaces with textured pillar and irregular surface features at micron length scales and at microsecond temporal resolution. The pillar superhydrophobic surfaces consisted of square micron-sized pillars spray coated with sub-micron PTFE particles while the irregular superhydrophobic surface was a nanocomposite coating. A drop was set in motion on these surfaces so that its three-phase receding contact line dynamics could be recorded using a high speed camera for qualitative and quantitative analysis.

## Methods

### Fabrication of the textured pillar surface

The fabrication of textured pillar surface pillars involved various steps: spin-coating of photoresist SU-8 3050 (Microchem. USA) on a silicon wafer, soft-baking of the material followed by UV exposure with mask aligner, post-exposure baking and finally washed for development^37^,^38^. Secondary roughness was then created by spray coating poly(tetrafluoroethylene) (PTFE) sub-micrometer particles, on the pillar tops. The concept of creating secondary roughness on top of a pillar surface for the creation of anti-wetting materials was also used by Gao *et al.*^23^, Steele *et al*.^39^ and Cao *et al*.^40^. The existence of these two length scales on the pillar surfaces have been shown to relieve receding contact line pinning^23^ and was hence utilized for this experiment.

**Figure 1:**
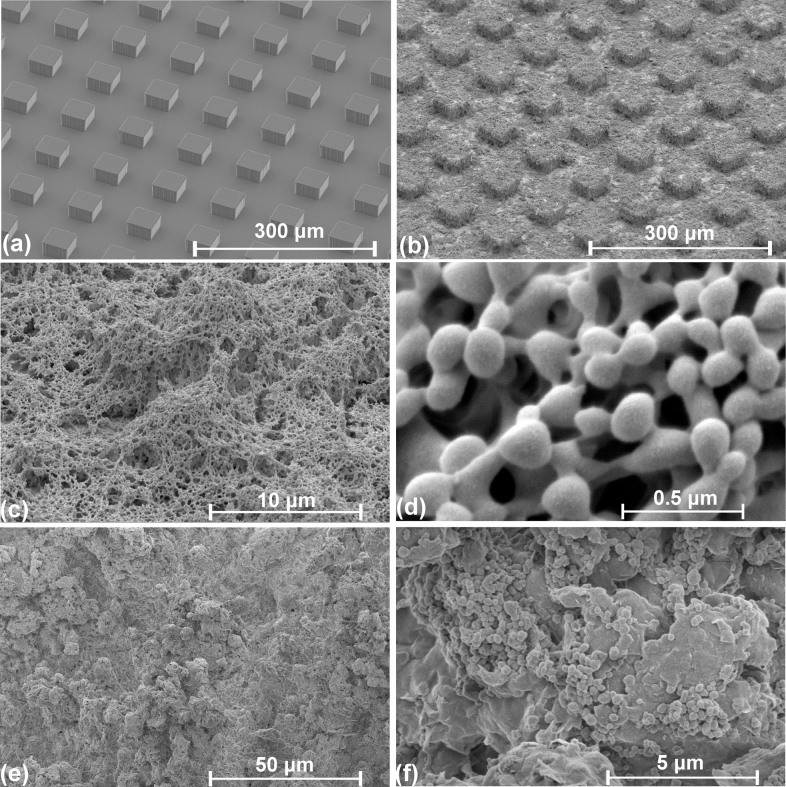
SEM images of a pillar surface at L = 63 μm and nanocomposite coating (a) pristine surface of pillar surface prior to deposition of PTFE particles (b) superhydrophobic pillar surface after deposition of PTFE particles (c) 5000× magnified image of the PTFE particles on a pillar and (d) 80,000× magnified image of PTFE particles on a pillar (e) 1000× and (f) 20,000× magnified image of nanocomposite coating, respectively.

The photoresist was first dispensed directly from the bottle onto a silicon wafer and spin-coated in two subsequent steps: (a) at 500 rpm for 10 s with spinning acceleration of 100 rpm/s and (b) at 4,000 rpm for 30 s with spinning acceleration of 300 rpm/s. The samples were then soft-baked at 100°C for 20 min on a hotplate, resulting in a film thickness of 33 μm. A soda lime mask of square-shaped patterns (42 μm) from Deltamask, Netherlands, at various inter-square distances of 63, 90, 105 and 120 μm were used for the exposure of the spin-coated samples. Patterning was performed by exposing the spin-coated material to UV radiation with a Karl-Suss MA6 mask aligner in hard contact mode with an i-line mercury lamp. An exposure dose of 323 mJ was used to fully polymerize the SU-8 layer. The exposure was followed by a post-exposure bake on a hotplate at 65°C for 1°min and at 95°C for 5 min, in order to achieve complete cross-linking of the resist. The samples were then allowed to cool down in order to improve adhesion of SU-8 to the silicon wafer. Subsequently, the samples were washed with a SU-8 developer followed by rinsing with 2-propanol. As a result, four samples consisting of 33 μm height, 42 μm width square pillar structures at 63, 90, 105 and 120 μm inter-pillar distances were obtained. A scanning electron microscope (SEM) image of a pristine 63 μm inter-pillar surface is shown in Figure 1a.

3% wt. PTFE particles (200–300 nm diameter) purchased from Sigma Aldrich were then dispersed in acetone via sonication and sprayed on top of the pristine SU-8-patterned pillars at approximately 10 cm with an air-assisting nozzle. This process introduced submicron/nanoscale roughness on top of the pillars and rendered the surface superhydrophobic. The impact of the PTFE particles on the surface during the spraying process also induces local electrostatic charging interactions between the particles and the substrates^41^. This causes Teflon particles to adhere to the surfaces. This is considered to be sufficient to hold the particles on the surfaces. The Scanning electron microscope (SEM) images of a surface (63 μm inter-pillar spacing) at pre- and post-spray are shown in Figure 1a and 1b, respectively. Note that the PTFE particles were also dispersed in the areas between the pillars (Figure 1b). Since the surface is in a Cassie wetting state, the water drop used in the experiment does not penetrate into these asperities and therefore the PTFE particles that were sprayed in these areas do not affect the contact line dynamics. SEM images of the submicron particles on top of a single pillar are also shown at two magnification levels in Figure 1c and 1d. The apparent equilibrium contact, advancing, receding and roll-off angles for the four pillar surfaces as well as for the unstructured superhydrophobic surface described in the next section were measured three times at three separate locations on the substrate using a goniometer (Model 250, Ramé-Hart, USA) with a 10 μL water drop and reported as averaged measurements with standard deviation as shown in Table 1. Results showed different correlations with the length of inter-pillar spacing (L). The averaged advancing angle remained independent of L and ranged between 160° to 167° for all pillar samples. However, the receding angle was found to increase with larger L distances. At a maximum L of 120 μm, the averaged receding angle was measured to be 144° as compared to 131° for an L of 63 μm. These correlations were consistent with results reported from previous experimental and computational studies^8^,^9^,^21^,^31^,^42^. In addition, the roll-off angle (ROA) of the surfaces were found to be inversely proportional to L, a relation that was in agreement with the force balance equation for a drop at an inclination prescribed by Yeh *et al*^8^. It should be noted that these apparent angles were measured using the tilt method and acquired at the incipient of drop motion, a measurement method that is widely recognized and accepted by researchers^43^.

### Fabrication of the nanocomposite surface

The irregular superhydrophobic surface was created by spray-casting precursor solutions on aluminum substrates followed by thermosetting to produce the final nanocomposite coatings. This method of nanocomposite fabrication was described in detail by Steele *et al*^44^. Nanocomposite surfaces are gaining prominence as they are robust, cost-effective and easy to apply on realistic large-scale applications^45^. This is compared to pillar surfaces which are fragile and often associated with expensive and restrictive fabrication processes.

First, 4 g of as-received dimethyl dialkyl C14–C18 amine functionalized montmorillonite clay particles (Nanoclay, Nanocor Inc., USA) were dispersed in 20 g of acetone. Waterborne fluorinated acrylic copolymer (25 wt% polymer, 75 wt% water; Capstone ST-110, DuPont., USA) was added slowly to the acetone-nanoclay suspension and blended with vortex mixing for 10 minutes, creating a Pickering emulsion. To create the nanocomposite coatings from this precursor solution, the slurries were spray-casted from 7.5 cm above the aluminum substrates and at 138 kPa (20 psi) using an internal-mix air-atomizing spray nozzle (1/4 JCO series, Spray Systems Co., USA) and then heat cured at 100°C for 8 hours. To ensure consistency in the spray-casting process and ultimately in the quality of the nanocomposite coatings, the aluminum substrate was spray coated in controlled longitudinal (Y axis) and lateral (X axis) motions driven by stepper motors (Xslide, Velmex Inc., USA). This automated spray-casting process was described in further detail by Yeong *et al*^46^. The resulting product from this process was an approximately 100 μm thick superhydrophobic nanocomposite coating depicting irregular but hierarchal surface features. This is seen in the SEM images shown in Figures 1e and 1f which revealed a surface texture at different length scales, with sub-micron sized features embedded within micron sized structures. The existence of this hierarchal structure not only promotes but stabilizes the superhydrophobicity of the coating as well^47^.

### Experimental set-up

**Figure 2:**
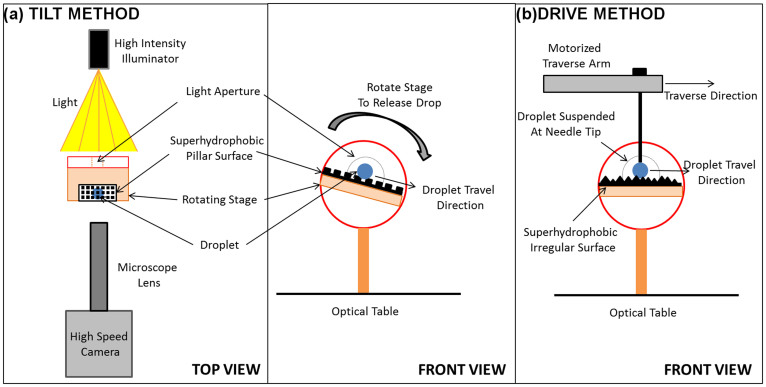
Schematic of the experimental setup depicting the (a) tilt method and (b) drive method.

Schematics of the experimental set-ups are shown in Figure 2. As shown in the figure, there were two methods in which the drop was advanced and receded across the superhydrophobic surfaces. The first method (Figure 2a) involved placing the textured pillar surface on a high precision rotation stage (PRM-1, Thorlabs, USA) and manually tilting it at an approximate rate of 3.5 degrees/s to allow a water drop (10 μL) to roll from the surface. High magnification optics coupled with high-speed imagery was utilized to record the dynamics of the microscopic receding angle while the droplet was traveling down the inclination. This was accomplished by attaching a microscope lens (6.5× UltraZoom fine focus with 2× F-mount adapter and 2× Lens attachment, Navitar, USA) to a high-speed camera (Fastcam SA-4, Photron, Japan). Under the back-lighting of a high intensity fiber optic illuminator (MI-150, Dolan-Jenner, USA) and through the aperture of the rotating stage, high contrast images of the microscopic receding contact line motions on each pillar were acquired at 15,000 frames/s (66 μs between each frame), with a resolution of 193 × 181 pixels for each frame. The entire set-up was constructed on top of an optical table to reduce external vibrations that could potentially introduce noise to the measurements. The contact line motions were recorded over 4 pillars for each of the four textured pillar surfaces of different inter-pillar distances. The recordings were then repeated at a different location on the substrate.

However, due to difficulty in acquiring acceptable image resolution on the nanocomposite coating with the tilt method, the drop was suspended at the tip of a needle which was attached to a motorized traverse arm (Xslide, Velmex Inc., USA) and driven horizontally across the surface. This is shown in Figure 2b. The speed of traverse was set at 5.8 cm/s and was prescribed based on preliminary measurements of the drop roll-off speed on the coating at tilt. Once the drop was in motion, the progression of the microscopic receding angle on the surface features were recorded using the same imaging techniques used for the pillar surfaces. This experimental technique involving a driven drop was also used in a recent receding angle study by Paxson and Varanasi^33^.

## Results and Discussion

### Textured pillar surface

**Figure 3:**
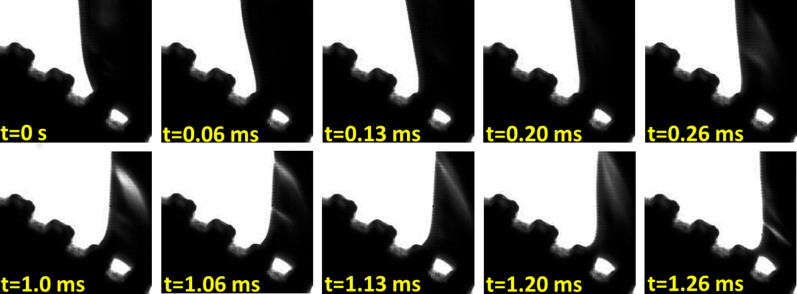
Sequence of high-speed images depicting the initial (0 to 0.26 ms shown in first row) and final events (1 ms to 1.26 ms shown in second row) of the microscopic receding line motion on a pillar surface (L = 63 μm) while at tilt. The total duration of the receding line motion on the pillar is 1.26 ms. The resolution for each frame is 193 × 181 pixels.

Figure 3 shows the images depicting the initial (0 to 0.26 ms shown in first row of Figure 3) and final events (1 to 1.26 ms shown in second row of Figure 3) of the receding line motion on a single pillar for a surface with an L value of 63 μm. The total duration of the receding line motion on the pillar is 1.26 ms. These images represent the key events during a single cycle of dynamic interaction between the receding line and the pillar. It can be observed that the receding line was initially (at t = 0 s) relaxed and had a high contact angle. However, it rapidly (within 0.13 ms) transitioned into a stage where the contact line was stretched and pulled inwards to form a concave shape as it traversed across the top of the pillar surface. The travel of the receding line between the duration of 0.26 ms and 1 ms was however limited, and only occurred over a small interface distance. Necking of the receding contact line would eventually start to occur after t = 1 ms with the formation of instabilities on areas of the drop located within close proximity to the pillars and the pinned contact line. These instabilities were due to drop vibrations triggered by the de-pinning process of the receding contact line. They were depicted as bright slit lines on the images and were formed as a result of light penetration from the back-lighting. The receding line was further stretched until the very last moment at t = 1.26 ms before the rupture and collapse of the capillary bridge. This caused the receding line to “snap” and advance to the adjacent pillar.

**Figure 4:**
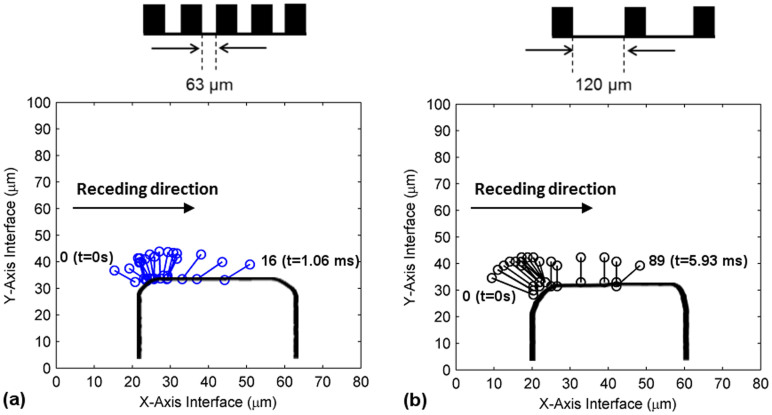
Traces of the positions of individual microscopic receding lines on a pillar from the receding line onset until point of “snapping” for surfaces (a) L = 63 μm and (b) L = 120 μm.

The dynamics of the receding line were analyzed in detail at the microscopic level by tracing it from the point where it intersects the pillar to a location along the length of the contact line situated approximately 10 μm away from the intersection. This was performed on all of the receding line images (recorded at a temporal resolution of 60 μs) that were acquired on a single pillar in the experiment. They were then reconstructed in a single plot and superimposed on a pillar outline to represent the onset, progression and ultimately “snapping” of the receding line on a pillar in a precise two dimensional space. This is shown in Figure 4a and 4b for pillar surfaces with L values of 63 and 120 μm, respectively. This deconstruction of the receding line motion provided detailed information about the variation of its microscopic three-phase angle as well as its spatial and temporal dynamics, all of which are comprehensively analyzed herein. It should be noted that the receding direction in the figure is to the right. The values labeled in the figure indicate the corresponding initial and pre-snap receding lines with the higher value representing the number of frames required to capture the complete receding line motion on a pillar. The time required for the receding line to travel from its initial to a pre-snap position was also labeled. It can be observed that the receding line remained much longer on the pillar surface where L = 120 μm (Figure 4b), as compared to the one at 63 μm (Figure 4a), an observation that will be addressed in the later parts of this discussion.

**Figure 5:**
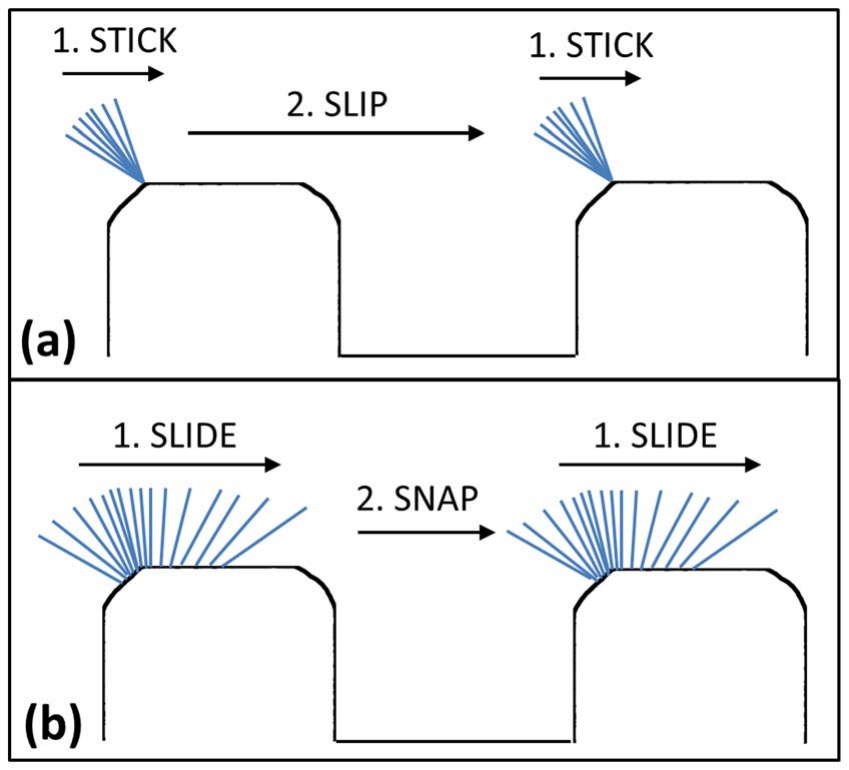
Schematic comparing the (a) “stick-slip” and (b) “slide-snap” dynamics of a microscopic receding line, with the arrows pointing in the direction of the contact line motion. The “stick-slip” dynamics was proposed by previous studies while the “slide-snap” dynamics was observed in the current study.

Spatial analysis of the receding line motions in Figure 4a and 4b revealed no substantial differences in its travel path between pillar surfaces of L = 63 μm and 120 μm. At the onset of the receding motion, the three-phase line was in contact with the lower left edge of the pillar, after which it would quickly travel around the edge to arrive at the pillar top surface. The receding contact line would then move horizontally along the length of the pillar top in small increments (approximately 1 μm per frame) for an extended period of time. This resulted in a receding line travel that was concentrated on a horizontal area close to the left edge of the pillar. The receding line would however abruptly accelerate to advance in larger increments and release itself from the pillar at approximately mid-distance between the left and right edges of the pillar top surface. Due to limitations in the temporal resolution of the camera, the precise location of receding line “snapping” could not be determined. There is a possibility that the microscopic receding line had further traveled (within the last 0.06 ms) to the right edge of the pillar before detachment at that location. In any case, there were substantial differences between the receding line spatial dynamics acquired in this experiment with hypotheses obtained from previous studies. Majority of the researchers have proposed a “stick-slip” motion of the microscopic receding line where the contact line would remain pinned (“stick”) at a specific point on the pillar before detaching (“slip”) to move to the next pillar^15^,^19^,^20^,^21^,^23^. However, based on current experimental observations, we propose that the receding dynamics of the microscopic receding line more closely resembled a “slide-snap” motion. Although Extrand^18^ did correctly predict the “sliding” motion of the receding contact line, it was suggested that the receding line would be pinned at the edge before being gradually pinched and ruptured. This was not observed in the current experiment. The differences between these two contact line dynamics are illustrated in a schematic shown in Figure 5.

**Figure 6:**
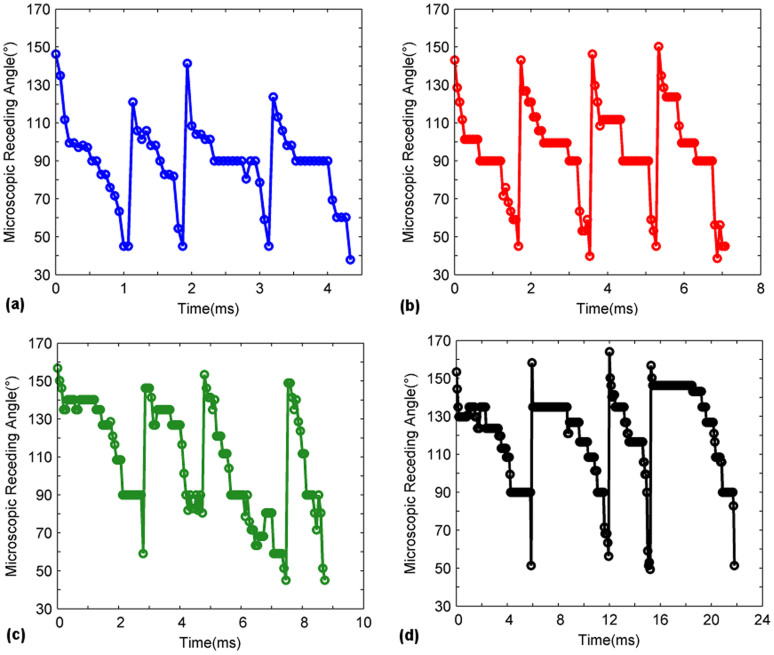
Measurements of the angles of each receding contact line as a function of time for a length of four pillars. Surfaces consist of (a) L = 63 μm (b) L = 120 μm (c) L = 105 μm and (d) L = 120 μm.

The microscopic receding line contact angles on the pillar surface as they progressed from one pillar to another were individually measured for four consecutive pillars and for all pillar surfaces (L = 63, 90, 105 and 120 μm). The angles were then plotted with respect to time as shown in Figure 6. The uncertainty of the angle measurements is estimated to be ±5°. Results show significant variation (90 degrees and above) between the angle measured at the onset of the receding motion (the maximum angle) and the angle measured right before the snapping of the receding line (the minimum angle). After the detachment of the receding line from the pillar, the angle of the contact line would abruptly increase as a new cycle of receding line motion commenced on the adjacent pillar. These receding angle dynamics were observed to be reasonably repeatable for each pillar and for all surfaces (L = 63–120 μm). Similar dynamics were observed when the measurement was repeated for a second time on four separate pillars on a different location on the substrates with different inter-pillar distances, i.e. a high receding angle at the onset, followed by a drop in the angles in accordance to the trends observed previously in the first measurement before detachment at a minimum angle. This shows that the measurements are repeatable. In addition, the microscopic receding line interface speeds of the first and second measurements were investigated will be addressed later on. The low angles (40–60° for all surfaces) of the pre-snap receding angles suggest a strong affinity of the liquid on the textured pillars. This is hypothesized to be due to the edge effects introduced by the textured pillars. This observation correlates with the study of Bhushan *et al.*^42^ who reported the pinning of a drop at the pillar edges. If the pillars were hydrophobically functionalized to produce a smooth top surface, the pre-snap receding angles can be expected to be higher.

**Figure 7:**
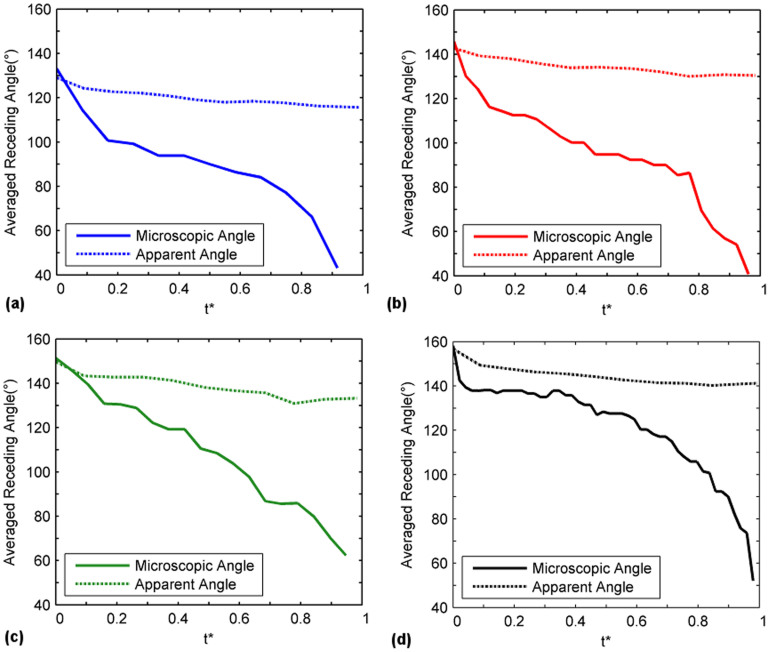
Averaged microscopic and apparent receding angles acquired for a distance of four pillars as a function of non-dimensional time (t* = (t − t_o_)/T) where t, t_o_ and T represents the current, initial and total duration of receding line travel on a single pillar, respectively.

The microscopic receding angles from all four pillars were averaged and plotted with respect to its non-dimensional time, t*, which was prescribed as (t − t_o_)/T, where t, t_o_ and T represents the current, initial and total duration of receding line travel on a single pillar, respectively. In addition, averaged apparent receding angles of the surfaces which were acquired at the apparent (millimeter) length scales were plotted with the microscopic results. This is shown in Figure 7 for all pillar surfaces. The differences in the receding angle between an apparent and microscopic measurement was observed to be substantial. While the initial measurements of the onset receding angle for the apparent and microscopic methods yielded similar values, it would however diverge as the receding line progressed across the pillar surface. This was to be expected; the initial receding angle did not involve any complex motions and could be measured without difficulty regardless of length scales. However, once progressed, the macroscopic field of view was insufficient to accurately capture the intricate motions of the three-phase line on each pillar, resulting in a divergence. Therefore, while the fluctuation of the angles from a microscopic measurement would exceed 90 degrees, the variation in apparent angles for all surfaces was consistently limited to within 20 degrees. The variation in magnitude of the apparent angles was consistent with previous studies at similar length scales^27^,^32^.

**Figure 8:**
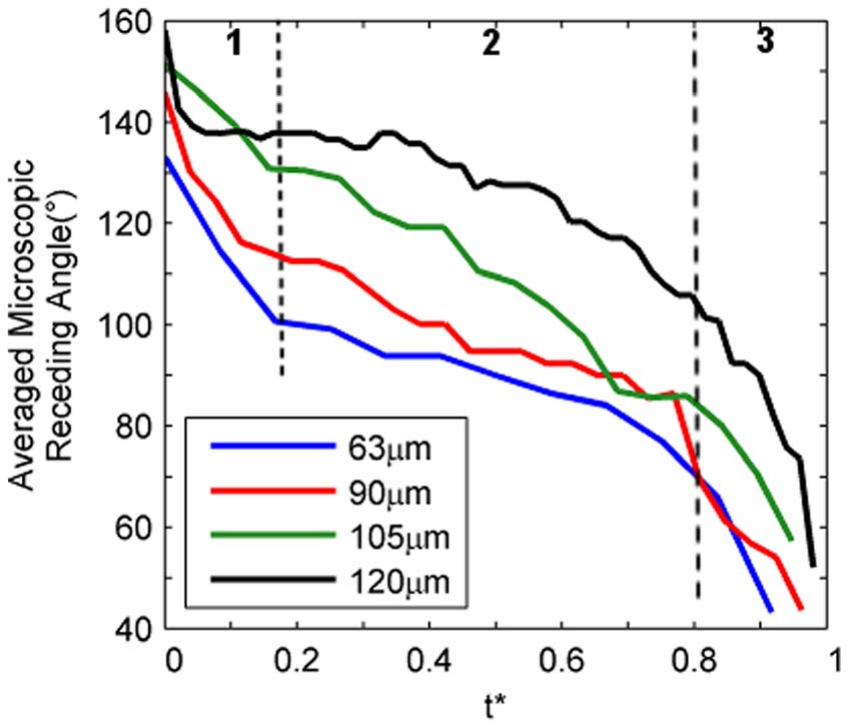
A comparison of the averaged microscopic receding angles for all pillar surfaces at varying L distances as a function of non-dimensional time (t* = (t − t_o_)/T) where t, t_o_ and T represents the current, initial and total duration of receding line travel on a single pillar, respectively.

A comparison of the averaged microscopic receding angles for all pillar surfaces at different L values was conducted and shown in Figure 8. The dynamics of the receding angles as it travelled on the pillar top to the point of detachment could be categorized into events that occurred in three time segments. These segments are labeled as areas 1 to 3 in Figure 8. In the first segment, the receding contact angle decreased at a high rate of descent. However, at the second time segment which constituted approximately 65% of the total duration of receding line travel, the decrease would be much more gradual. Once in the third and final segment, the receding angles would sharply decrease again until the point of de-pinning. It should be noted these time segments corresponded with the occurrence of the three receding line motion characteristics previously described for Figure 4a and 4b, i.e. a quick receding line travel around the left edge of the pillar, a short interface traverse distance on the pillar top and an abrupt receding line advancement in larger spatial increments until the point of detachment. In addition, it could be observed that as L increased, the curve line shifted upwards, resulting in larger initial receding angles (at T* = 0). The pre-snap receding angles (T* > 0.9) also showed a tendency to increase with L. This was caused by the length of the capillary bridge. For smaller L values, the shorter capillary bridge interacts with its adjacent capillary bridges and affects the deformation of the receding line^33^. This meant that the initial receding line on a pillar was unable to relax, and therefore had lower receding angles. This is in contrast to when pillars were spaced further apart and where interactions between pillars were kept to the minimum, which led to higher receding angles at the beginning of receding motion. A similar explanation could be made for the pre-snap receding lines; Receding lines were able to detach at larger angles at larger L's, as compared to being stretched to a lower angle when under the influence of a nearby capillary bridge^31^. As previously mentioned, the apparent receding angles (Table 1) were also found to be linearly proportional to L, albeit at different magnitudes.

**Figure 9:**
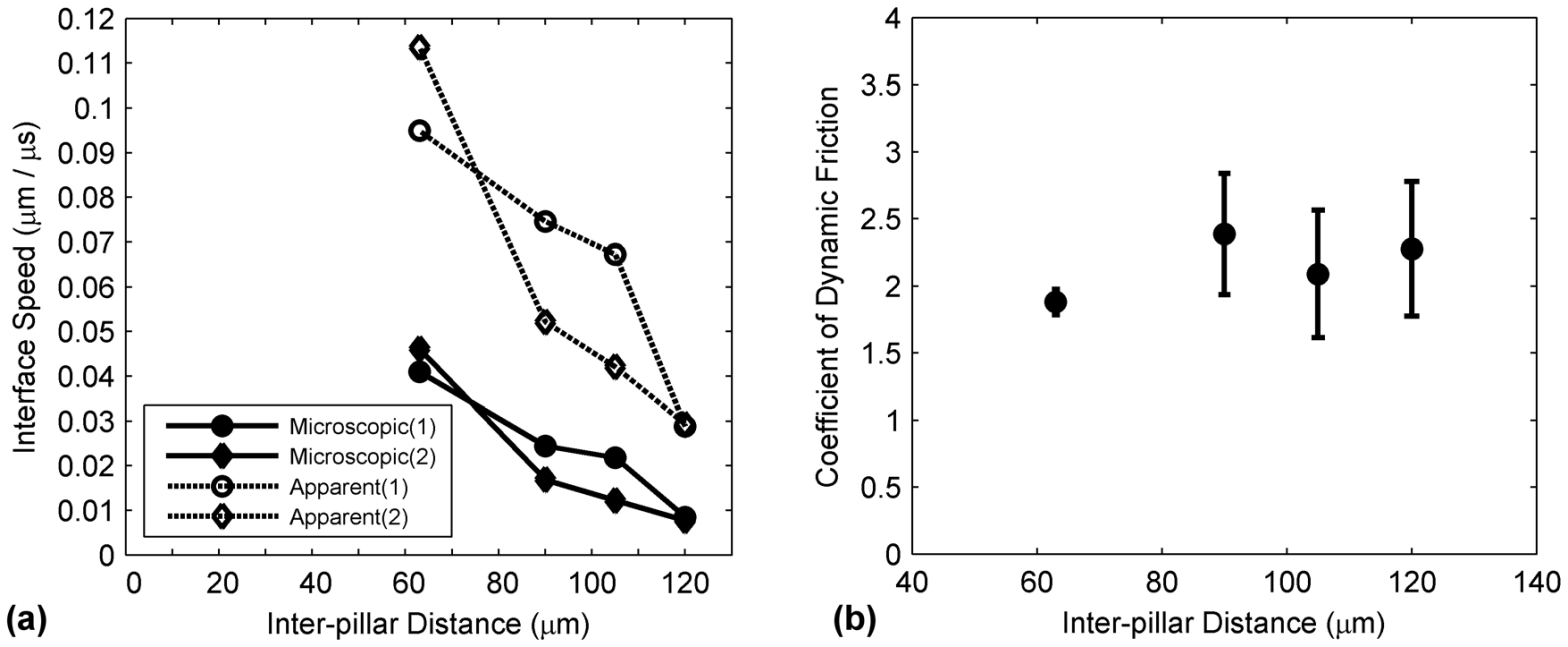
(a) Averaged travel speed of the apparent and microscopic receding lines as a function of inter-pillar distance. The measurements were repeated twice with the order of measurement included in the parenthesis of the legend. (b) Coefficient of dynamic friction of pillar surfaces calculated from data shown in Table 1.

A measurement of the interface travel speed for apparent (macroscopic) and microscopic receding lines for all pillar surfaces was performed. These two travel speeds are defined in Equation 1 and 2.
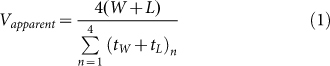

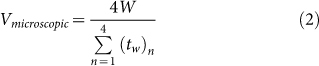
where *W* is the width of the pillar, *L* is the inter-pillar distance, *t* the time required to traverse a distance of either the width of the pillar (denoted by the subscript *W*) or the inter-pillar distance (denoted by the subscript *L*). In addition, the subscript *n* refers to the number of pillars that the drop has travelled on. Therefore, the apparent interface travel speed was evaluated over a distance of 4 pillar widths and inter-pillar distances which span a total distance between 424 and 652 μm for all test surfaces while the microscopic interface travel speed was based on the average time required to travel a single pillar width (43 μm). It can be observed in Figure 9a that regardless of the inter-pillar distance, the values *V_apparent_* are higher than *V_microscopic_*. This is due to the fact that *t_L_*, which refers to the time taken for the contact line to travel the inter-pillar distance, or the time required for the contact line to “snap” to its adjacent pillar, occurs very quickly at less than 0.067 ms. Hence, the contribution of this term to Equation 1 can be considered to be negligible. This results in a larger value for *V_apparent_* as compared to *V_microscopic_*. This observation reaffirms the fact that the dynamics of receding contact lines at the macroscopic level differ from those at the microscopic scale. In addition, results in Figure 9a show a similar trend for both apparent and microscopic measurements, i.e., a decrease in interface speed with increasing L, albeit at different magnitudes. This was caused by the shorter duration of travel on a pillar with small L values, as compared to a longer travel duration on a pillar with large L values. This was previously observed in Figures 4a and 4b and could be attributed to the tangential component of the gravity force that is exerted on the drop. As reported in Table 1, the ROA of the L = 63 μm surface is approximately a factor of two of the ROA of an L = 120 μm surface. As such, the drop on the L = 63 μm surface was subjected to a stronger downward force and therefore released from the surface at higher speeds. It should also be noted that the repeated measurements of interface speed (for both apparent and microscopic) shown in Figure 9a compare favorably with the measurements acquired the first time. The slight differences between the two measurements can be attributed to the variance in the surface ROA as indicated by the values in Table 1.

The ROA effect could also be shown by considering the force balance equation of a drop on a superhydrophobic surface described in Equation 3 where the tangential force exerted on the drop is balanced with its frictional force.
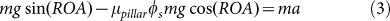
where *m* is the mass of the drop, *g* is gravity, *μ_pillar_* is the coefficient of dynamic friction, *φ_s_* is the solid area fraction of the surface, and *a* is the acceleration of the drop^48^. The solid area fraction is defined in Equation 4^8^.
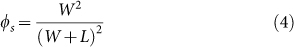


Since the measurements were performed at the onset of drop rolling, the acceleration of the drop was considered to be negligible. This was shown in Figure 6, where the angles of each receding contact line were plotted as a function of time. It can be observed that the duration required for the contact line to travel across each pillar for all L cases were approximately constant. This was also reported by Sakai *et al.*, where drop rolling velocity from 0 (point of release) to 20 ms was measured to constant^48^. However, acceleration of the drop was measured by Sakai *et al*. at longer drop rolling times. Since the current study did not include imaging past 4 pillars, this observation could not be confirmed. Hence, with the negligible acceleration assumption, Equation 3 was then reduced to describe the coefficient of dynamic friction as a function of ROA and solid area fraction. This is shown in Equation 5.
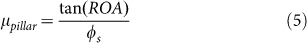


It should be noted that at longer drop travel times where the acceleration of the drop is anticipated to be more apparent, the acceleration term in Equation 3 will have to be taken account and thus affect the analysis of the equation. Equation 5 was evaluated for all pillar surfaces and results were plotted in Figure 9b. Results show that the coefficient of dynamic friction experienced by the drop for all pillar surfaces, based on measurements at the onset of drop rolling, is approximately constant. Therefore, this indicates that a surface with a decreasing solid area fraction will be accompanied by a decreasing ROA as well, an observation which is consistent with the trends that were measured in the experiment.

### Irregular nanocomposite surface

**Figure 10:**
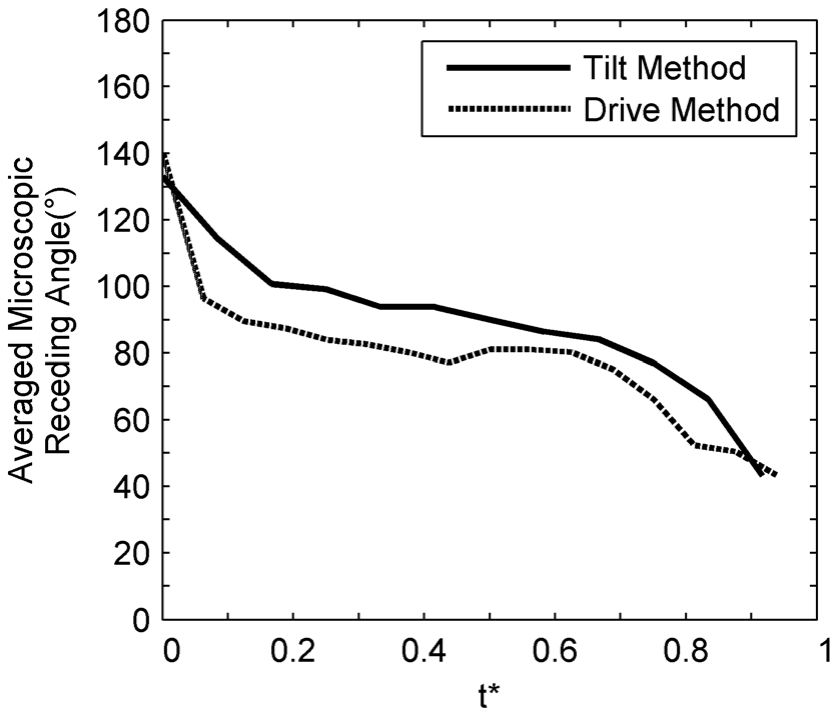
A comparison of the averaged microscopic receding angles acquired with the tilt and drive method on a pillar surface with 63 μm inter-pillar spacing as a function of non-dimensional time (t*). Results show that measurements from the tilt and drive experimental technique compare favorably.

The study of microscopic receding line motion was extended on a nanocomposite surface with irregular, hierarchal surface features at the micro- and nano- length scales using the drive method as previously described in the experimental methods section. A separate study was conducted to examine the differences between the tilt and drive methods. The microscopic receding angles on a pillar surface with inter-pillar distance of 63 μm was measured with the drive method and compared with measurements previously obtained with the tilt method. The examined parameter is the averaged microscopic receding angles for four pillars as a function of non-dimensional time t*, similar to Figures 7 and 8. Results from Figure 10 showed that the angles from the tilt method compares reasonably well with the drive method. The initial receding (at t* = 0) and pre-snap (t* > 0.9) angles from both test methods compares favorably. In addition, a similar trend of rapidly decreasing receding angle on the pillars over time was observed. This shows that the drive method can be used as an alternative experimental method to measure the dynamics of the microscopic receding angles.

**Figure 11:**
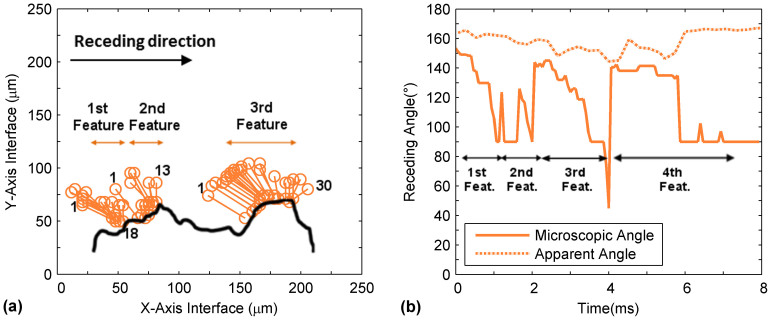
(a) Sketches of the position of individual microscopic receding lines on an irregular nanocomposite coating from the receding line onset until point of “snapping” (b) measurements of the angles of each receding contact line as a function of time for a length of four irregular surface features.

An analysis of the deconstructed receding lines on the surface features similar to Figure 4 was performed with results shown in Figure 11. As with the earlier investigation on pillar surfaces, the receding direction was to the right. Since the features did not consist of repeatable patterns like the pillars, they were specified based on the locations of initial and pre-snap receding lines which were labeled accordingly in the figure. We observed general similarities of the receding line motion of this irregular surface with the pillar surfaces. The occurrence of “slide-snap” was preserved. Distinct angle variations of the microscopic receding line as it slid on a feature were also observed. This is shown in Figure 11a.

However, some differences were noted. For example, the distance of travel of the receding line on the first and second feature was less pronounced as compared to the case of pillar surfaces. Moreover, the pre-snap receding angles for these features were measured to be much higher at 90 degrees. (Figure 11b) The reason for this difference was attributed to the shape and contact area of the features. Surface structures that exhibit distinct pointed ends will have a smaller exposed area for wetting and attachment and therefore will depict receding motion at a localized area with higher angles right before detachment. This corresponds with the hypotheses from Krumpfer *et al.*^20^ and Priest *et al.*^28^ who predicted a high receding angle outcome for surfaces with needle-like structures or posts having conical tops. This discovery was further validated by comparing these receding dynamics to the third feature shown in Figure 11a. The structure of this feature consisted of a flatter top and had a width of approximately 40 μm, hence bearing physical resemblance to the pillar surfaces that were previously investigated. It could be observed that its receding line dynamics were consistent with measurements that were previously acquired, i.e. the sliding of the receding line across the horizontal top of the feature followed by a detachment at 45 degrees. Therefore, this confirms the influence of the feature structure on the microscopic receding angle dynamics.

## Conclusions

The study of microscopic receding line motion acquired at microsecond time resolution on pillar and irregular nanocomposite superhydrophobic surfaces revealed contact line dynamics that were previously not reported. The receding line progressed from the lower edge of a pillar, across the length of the pillar top before “snapping” to advance to the adjacent pillar, creating a “slide-snap” motion. This is in contrast to the “stick-slip” motion that was reported in previous studies. The variation of the microscopic receding angle for this entire sequence of motion was measured to be significant with a difference of approximately 90 degrees between the angles measured at the initial and pre-snap of the receding line. Similar measurements performed at the macroscopic level would only yield a difference of approximately 20 degrees. This observation was consistent for all investigated pillar surfaces with varying L distances. The apparent and microscopic interface travel speeds were both found to be inversely proportional to L. This was due to the fact that the roll-off angles for surfaces with smaller L values were larger which resulted in a stronger exertion of tangential gravity force on the drop. Similar experiments performed on a nanocomposite surface revealed a similar “slide-snap” motion of the microscopic receding line. However, due to the sharper features of the surface, the angle of the receding line prior to detachment was measured to be higher (at 90 degrees) as compared to the pillar surfaces.

## Author Contributions

Y.H.Y. ran the experiments, wrote the main manuscript text and prepared figures. A.M. prepared all superhydrophobic samples used in this study and prepared Figure 1. E.L. and I.B. reviewed the manuscript.

## Figures and Tables

**Table 1:** Averaged apparent measurements of the superhydrophobic performance of the pillar and nanocomposite surfaces with standard deviation

Type	Interpillar Distance (μm)	Contact Angle(°)	Advancing Angle(°)	Receding Angle(°)	Roll-Off Angle(°)
Textured Pillar	63	152.0 ± 2.8	166.6 ± 3.3	131.5 ± 4.4	17.2 ± 0.9
Textured Pillar	90	161.4 ± 1.0	160.2 ± 1.9	134.5 ± 2.6	14.0 ± 2.7
Textured Pillar	105	160.1 ± 1.5	167.3 ± 0.9	140.9 ± 2.9	10.6 ± 2.3
Textured Pillar	120	157.3 ± 2.2	165.3 ± 0.9	144.3 ± 3.1	9.1 ± 2.0
Irregular	N/A	155.6 ± 1.5	156.2 ± 0.8	146.9 ± 2.5	3.1 ± 0.5
